# The complete chloroplast genome of *Morus alba* (Moraceae: Morus), the herbal medicine species in China

**DOI:** 10.1080/23802359.2019.1638328

**Published:** 2019-07-13

**Authors:** Jiang Luo, Yingying Wang, Allan ZiJian Zhao

**Affiliations:** a Jiangxi Province Hospital of Integrated Chinese and Western Medicine, Nanchang, Jiangxi, China;; b School of Ophthalmology and Optometry, School of Biomedical Engineering, Wenzhou Medical University, Wenzhou, Zhejiang, China

**Keywords:** *Morus alba*, Moraceae, herbal medicine, chloroplast genome, phylogenetic relationship

## Abstract

*Morus alba* is commonly called white mulberry, which is native to China and is the tender branch of mulberry as a herbal medicine in China. In this study, we presented and annotated the complete chloroplast genome of *M. alba*. The whole chloroplast genome is 159,050 bp in size, exhibiting a large single copy region (87,762 bp), a small single-copy region (19,876 bp) and a pair of inverted-repeat regions (25,706 bp). The overall nucleotide composition is: 31.4% of A, 32.4% of T, 18.4% C, and 17.8% G, with a total A + T content of the chloroplast genome 63.8% and G + C content of 36.2%. The whole chloroplast genome of *M. alba* contains 126 genes, including 82 protein-coding genes (PCG), 36 transfer RNA (tRNAs), and 8 ribosome RNA (rRNAs). Phylogenetic Maximum-Likelihood (ML) tree based on 15 species chloroplast genomes that *Morus alba* is closely related to *Morus cathayana.* This complete chloroplast genomes can be used for medicinal value and clinical drug development that also can have a great significance for future to continue research.

*Morus alba* is the most important Moraceae species and commonly called mulberry, which is native to China and is widely cultivated and naturalized elsewhere. Branches, leaves, fruits, and barks of *M. alba* constantly can be used as a herbal medicine to treat fever, improve eyesight, strengthen joints, and lower blood pressure in China (Eric et al. 2016). It is generally a short-lived tree with a lifespan comparable to that of humans, although there are some specimens known to have been over 250 years old. The branches of *M. alba* have been used in China since at least 2500 B.C. as the primary diet for silkworms used to make silk, that greatly influenced world history through the Silk Road. It has multiple uses in pharmaceuticals and ecology, which has also been planted in various areas for erosion control and windbreaks all over the world (He et al. 2013). Simultaneously, rare genomic data was reported about *M. alba*, even the genus of Morus. In order to further study the *M. alba* and the Moraceae family genetic diversity and genetic structure of natural populations, we presented the complete chloroplast genome of *M. alba* and studied the phylogenetic relationship, which can be used for medicinal value and clinical drug development in future.

The specimen sample of *Morus alba* was collected from Jiangxi Province Hospital of Integrated Chinese and Western Medicine (Nanchang, Jiangxi, China, 115.91E; 28.67N). Total genomic DNA of *M. alba* was extracted from the branches tissue using Plant Tissues Genomic DNA Extraction Kit (Solarbio, BJ, and CN) and stored in Jiangxi Province Hospital of Integrated Chinese and Western Medicine (No. JXPHICWM02). The chloroplast (cp) DNA was purified and fragmented using the NEB Next Ultra^TM^ II DNA Library Prep Kit (NEB, BJ, and CN), after that the cpDNA was sequenced. Quality control was performed to remove low-quality reads and adapters using the FastQC software (Andrews 2015). The chloroplast genome was assembled and annotated using the MitoZ software (Meng et al. 2019). The physical map of the chloroplast genome was generated using OrganellarGenomeDRAW (Lohse et al. 2013). The accurate new annotated chloroplast genome was submitted to GenBank with accession No. MK8098921.

The whole complete chloroplast genome of *Morus alba* is with 159,050 base pairs (bp) in length as the circular, exhibiting a characteristic quadripartite structure with a large single-copy region (LSC) of 87,762 bp, a small single-copy region (SSC) of 19,876 bp and two inverted repeat regions (IRs) of 25,706 bp. The cpDNA of *M. alba* contains 126 genes, including 82 protein-coding genes (PCG), 36 transfer RNA genes (tRNAs), and 8 ribosomal RNA genes (rRNAs). In the IR regions, a total of 20 genes were found duplicated, including 9 PCG species (*rpl2, rpl23, ycf2, ndhB, rps7, rps12, ycf15, ycf68, a*nd *ycf1*), 7 tRNA species (*trnI-CAU, trnL-CAA, trnV-GAC, trnI-GAU, trnA-UGC, trnR-ACG* and *trnN-GUU*) and 4 rRNA species (*rRNA16, rRNA23, rRNA4.5,* and *rRNA5*). The overall nucleotide composition is: 31.4% of A, 32.4% of T, 18.4% of C and 17.8% of G, with a total A + T content of 63.8% and G + C content of 36.2%.

In Maximum-Likelihood (ML) analysis phylogenetic relationship, we selected other 14 of the family Ulmaceae and Moraceae species chloroplast genomes from GenBank to assess the relationship of *Morus alba*. The phylogenetic tree was reconstructed using the Maximum-Likelihood (ML) method. ML analysis was performed using the MEGA X software (Kumar et al. 2018) with 2,000 bootstrap values replicate at each node based on GTR model (Kumar et al. 2018). All of the nodes were inferred with strong support by the ML methods. The final tree was represented using the MEGA X software (Kumar et al. 2018) and edited using the iTOL 4.0 online web (https://itol.embl.de/) (Letunic and Bork 2016). Phylogenetic ML tree (Figure 1) analysis showed that the chloroplast genome of *Morus alba* is clustered and closest to *Morus cathayana* (GenBank No. NC_031822.1) in the phylogenetic relationship. However, the complete chloroplast of *Morus alba* is very important for the conservation and evolutionary studies of this species and also can be used for medicinal value and clinical drug development in the future.

**Figure 1.**
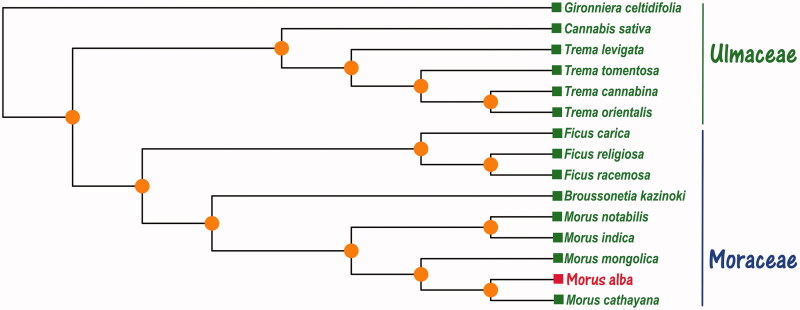
The Maximum-Likelihood (ML) phylogenetic tree was constructed and based on the complete chloroplast genomes data of the 15 family Ulmaceae and Moraceae. The GenBank accession numbers are as follows: *Broussonetia kazinoki NC_037021.1, Cannabis sativa NC_026562.1, Ficus carica NC_035237.1, Ficus racemosa NC_028185.1, Ficus religiosa NC_033979.1, Gironniera celtidifolia KY931662.1, Morus cathayana NC_031822.1, Morus indica DQ226511.1, Morus mongolica NC_025772.2, Morus notabilis NC_027110.1, Trema cannabina KJ687875.1, Trema levigata KY931666.1, Trema orientalis KY931667.1, Trema tomentosa KY931669.1.*
